# Cigarettes Smoking and Skin: A Comparison Study of the Biophysical Properties of Skin in Smokers and Non-Smokers

**Published:** 2019-02

**Authors:** Taraneh Yazdanparast, Hournaz Hassanzadeh, Saman Ahmad Nasrollahi, Seyed Mohammad Seyedmehdi, Hamidreza Jamaati, Amirkia Naimian, Maryam Karimi, Rahim Roozbahani, Alireza Firooz

**Affiliations:** 1 Telemedicine Research Center, National Research Institute of Tuberculosis and Lung Diseases (NRITLD), Shahid Beheshti University of Medical Sciences, Tehran, Iran,; 2 Pharmaceutical, Cosmeceutical and Hygienic Clinical Evaluation Laboratory (DermaLab), Center for Research and Training in Skin Diseases and Leprosy, Tehran University of Medical Sciences, Tehran, Iran,; 3 Chronic Respiratory Diseases Research Center, NRITLD, Shahid Beheshti University of Medical Sciences, Air Pollution, Health and Occupational Diseases Research Unit, Tehran, Iran,; 4 Clinical Tuberculosis and Epidemiology Research Center, NRITLD, Shahid Beheshti University of Medical Sciences, Tehran, Iran

**Keywords:** Biophysical properties, Skin, Smokers, Non-smokers

## Abstract

**Background:**

Tobacco smoke is toxic for cells and could be a damaging factor to skin. The purpose of this study was to compare the biophysical properties of skin in smokers and non-smokers.

**Materials and Methods:**

The study population consisted of 28 current smokers and 24 non-smokers. The hydration of the stratum corneum, trans epidermal water loss, pH, erythema, melanin content, sebum, friction and elasticity parameters (R0, R2, R5) of skin, epidermis and dermis thickness and echo-density were measured on middle forehead, right cheek and right inner arm of participants. Also volume, surface area and depth of right nasolabial folds were measured. The mean of these values in smokers were compared with non-smokers by independent sample T- test.

**Results:**

Gross elasticity was significantly lower in smokers on forehead (p= 0.048). Thickness of epidermis was higher in smokers in all measured sites but the differences were not statistically significant. Thickness of dermis was higher in smokers in all measured sites too, but only the difference on cheek was statistically significant (p= 0.009). Density of epidermis was lower in smokers in all measured sites, but only the difference on forehead was statistically significant (p= 0.019). Density of dermis was lower in smokers in all measured sites, but only the difference on arm was statistically significant (p= 0.028). Volume and area of nasolabial folds were higher in smokers, but only the difference of area was statistically significant (p = 0.031).

**Conclusion:**

Tobacco smoking could affect the biophysical parameters of skin, especially thickness and density of dermis and epidermis and nasolabial folds.

## INTRODUCTION

Tobacco smoke is toxic for cells and could be a damaging factor to skin. The various health effects of smoking are well known, but the effect of smoking on skin is less studied. It is shown that cigarette smoking increases many symptoms associated with aging process including skin symptoms and induces premature aging of skin (1, 2). The mechanisms responsible for effects of smoking on skin are not completely recognized.

The association of smoking with skin cancers is not clear yet and the results of different studies are contradictory (3–6). Some chronic dermatoses such as, contact and atopic dermatitis, psoriasis, and cutaneous lupus erythematosus have shown association with cigarette smoking. On the other hand, nicotine has been shown to be effective as a treatment in some skin diseases (7), and there has been no association between cigarette smoking and acne (8).

The effect of smoking on biophysical properties of skin has been studied. It has also been shown that skin color lightens after cessation of smoking (9), and both erythema and melanin indices of skin were reduced 1 month after cigarette cessation (10). Hemoglobin level of smokers was higher than the non-smokers (11) and nicotine was able to stimulate the activity of melanocytes (12, 13). The thickness of Stratum Corneum (SC) correlated negatively to the number of years of smoking (14) and the thickness of smokers’ skin was increased on the cheek, but not on other sites (15). Also, it is shown that smoking affects nasolabial folds and other coarse wrinkles of face (16).

Yin et al. showed tobacco smoking was an independent risk factor for the development of wrinkles and mentioned the need for objective and reliable methods for evaluating the biophysical properties of skin (17). So the purpose of this study was to compare several biophysical properties of skin in smokers with non-smokers in order to evaluate the effect of smoking on skin.

## MATERIALS AND METHODS

This analytical cross-sectional study was conducted with the cooperation of Research Institute of Tuberculosis and Lung Diseases, Dr. Masih Daneshvari Hospital and Center for Research & Training in Skin Diseases & Leprosy (CRTSDL). The study protocol was approved by CRTSDL institutional review board and Tehran University of Medical Sciences Ethics Committee. According to the Declaration of Helsinki, ethical considerations such as voluntary participation, participant informed consent, and confidentiality were respected.

The study population consisted of 52 volunteer men, including 28 current smokers and 24 never-smokers.

The exclusion criteria were history of average sun exposure more than 30 hours per month, any type of active skin diseases (such as dermatitis, psoriasis, skin cancers, etc), any systemic diseases that can affect skin status, and any face rejuvenation procedures. Passive smoking was an exclusion criterion only for non-smoker group.

To perform the biophysical assessments, participants were instructed not to use any topical products on their skin from the night prior to assessments. On the day of measurements, participants were asked to rest and relax for 20 minutes in the standard atmosphere (20–25 °C; 25 +/− 5% humidity) and then the hydration of the SC (using Corneometer® CM 825), Trans Epidermal Water Loss (TEWL) (using Tewameter® TM 300 device), pH (using Skin-pH-Meter® PH 905 device), erythema and melanin content (using Mexameter® MX 18 device), sebum (using Sebumeter® SM 815 device), friction value (using Frictiometer FR700 device) and skin elasticity parameters including R0, R2, R5 (using Cutometer® 580 device) were measured by Multi Probe Adapter (MPA, Courage + Khazaka electronic GmbH, Germany) on middle forehead, right cheek and right inner arm of participants, while they were in supine position.

Frictiometer measures the torque as friction index and the value is related to elasticity and plasticity of skin. Among elasticity parameters, R0 shows total elastic and plastic deformation of skin and is opposed to firmness, R2 shows gross elasticity, and R5 shows net elasticity (18).

Epidermis and dermis echo-density and thickness were measured by 22 MHz DUB skin scanner (TPM Company, Germany). These measurements were done on middle forehead, right cheek and right inner arm of participants, too.

Also volume, area and depth of right nasolabial fold of participants were measured by CSI software (Courage + Khazaka electronic GmbH, Germany) from their standard photographs.

The mean of these values in smokers were compared with non-smokers by independent samples T- test and the statistical significance level was defined as P≤0.05.

## RESULTS

The mean age of smokers and non-smokers were 37.68 (SD=11.14) and 36.29 years (SD=10.82), respectively. The minimum age was 23 and the maximum age was 63 years. The average years of smoking was 15.64 (SD=10.22) years and the average number of cigarettes smoked per day was 15.11 (SD=7.00) in smoker group.

The biophysical parameters of skin which have been assessed by MPA are shown in tables 1 (middle forehead), 2 (right cheek) and 3 (right arm). The SC hydration, skin pH and R0 (≠ firmness) were lower in smokers compared with non-smokers in all measured sites, but the differences were not statistically significant. The TEWL and melanin content in smokers were higher than non-smokers in all measured sites, but the differences were not statistically significant. Gross elasticity (R2) was significantly lower in smokers only on forehead (p= 0.048).

**Table 1.** Skin biophysical parameters on middle forehead of smokers and non-smokers (control group)

**Parameter (unit)**	**Smoker group Mean ± SD**	**Control group Mean ± SD**	**p-value** (independent sample-T test)
Hydration (arbitrary)	62.91±16.84	67.46±11.19	0.26
TEWL (g/m^2^/h )	16.01±13.87	13.01±4.82	0.31
Friction (arbitrary)	539.84±173.82	611.74±193.90	0.17
pH (arbitrary)	4.88±0.55	5.01±0.52	0.40
Sebum (μg/cm^2^)	80.46±38.42	106.33±64.78	0.09
Melanin content (arbitrary)	242.16±70.35	222.58±42.50	0.22
Erythema (arbitrary)	497.38±94.63	499.02±62.18	0.94
R0 (arbitrary)	0.218±0.071	0.230±0.085	0.59
R2 (arbitrary)	0.631±0.162	0.713±0.120	0.04
R5 (arbitrary)	0.332±0.088	0.355±0.159	0.53
Epidermis thickness (μm)	162.68±33.54	147.74±41.86	0.16
Epidermis density	101.16±40.05	124.13±23.34	0.01
Dermis thickness (μm)	2127.32±255.63	2083.22±300.27	0.57
Dermis density	16.44±24.13	18.48±9.76	0.70

SD = standard deviation

The echo-density and thickness of epidermis and dermis in both smokers and non-smokers groups are shown in tables 1 to 3. The thickness of epidermis was higher in smokers in all measured sites, but the differences were not statistically significant. The thickness of dermis was also higher in smokers in all measured sites, but only the difference on cheek was statistically significant (p=0.009). The echo-density of epidermis was lower in smokers in all measured sites, but only the difference on forehead was statistically significant (p=0.019). The density of dermis was also lower in smokers in all measured sites, but only the difference on arm was statistically significant (p= 0.028).

**Table 2.** Skin biophysical parameters on right cheek of smokers and non-smokers (control group)

**Parameter (unit)**	**Smoker group Mean ± SD**	**Control group Mean ± SD**	**p-value** (independent sample-+T test)
Hydration (arbitrary)	63.25±15.03	63.90±10.90	0.86
TEWL (g/m^2^/h )	15.19±8.77	14.70±5.89	0.81
Friction (arbitrary)	557.33±230.06	536.88±169.23	0.72
pH (arbitrary)	5.00±0.45	5.11±0.48	0.44
Sebum (μg/cm^2^)	110.64±93.04	105.95±55.17	0.83
Melanin content (arbitrary)	222.47±72.62	206.26±33.11	0.29
Erythema (arbitrary)	510.90±47.69	506.24±58.34	0.75
R0 (arbitrary)	0.192±0.076	0.216±0.074	0.28
R2 (arbitrary)	0.664±0.076	0.662±0.112	0.92
R5 (arbitrary)	0.428±0.145	0.392±0.155	0.39
Epidermis thickness (μm)	183.54±112.76	143.32±32.72	0.11
Epidermis density	102.40±21.30	112.27±37.24	0.27
Dermis thickness (μm)	2361.54±337.08	2105.95±320.18	0.00
Dermis density	13.68±7.13	18.50±12.93	0.12

SD = standard deviation

**Table 3.** Skin biophysical parameters on right inner arm of smokers and non-smokers (control group)

**Parameter (unit)**	**Smoker group Mean ± SD**	**Control group Mean ± SD**	**p-value** (independent sample-T test)
Hydration (arbitrary)	60.70±15.78	68.00±11.97	0.07
TEWL (g/m^2^/h )	7.83±13.89	6.39±2.95	0.62
Friction (arbitrary)	295.71±179.09	359.15±198.70	0.23
pH (arbitrary)	4.89±0.41	4.94±0.41	0.65
Sebum (μ g/cm^2^)	20.85±44.63	11.95±15.27	0.32
Melanin content (arbitrary)	163.54±43.46	157.37±28.96	0.55
Erythema (arbitrary)	271.73±53.29	265.37±53.34	0.67
R0 (arbitrary)	0.282±0.100	0.284±0.099	0.93
R2 (arbitrary)	0.875±0.047	0.885±0.051	0.48
R5 (arbitrary)	0.608±0.168	0.591±0.230	0.76
Epidermis thickness (μm)	137.64±15.14	131.73±22.72	0.27
Epidermis density	127.00±31.72	139.78±28.83	0.14
Dermis thickness (μm)	1159.60±421.90	1050.39±209.30	0.23
Dermis density	58.77±19.38	72.65±24.27	0.02

SD = standard deviation

**Figure 1.**
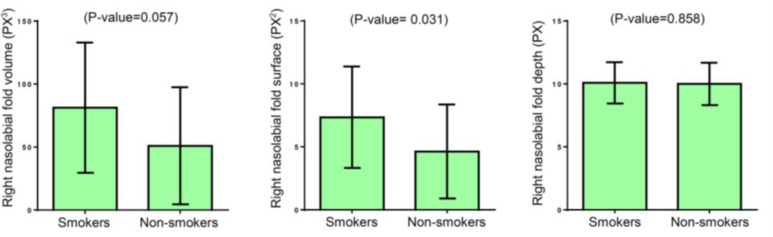
Measurements of right nasolabial fold characteristics by CSI software in smokers and non-smokers (control group), Error bars: standard deviation

Figure 1 shows that the volume, surface, and depth of right nasolabial fold were higher in smokers compared to non-smokers, but the difference in surface was only significant (p=0.031).

## DISCUSSION

The results of our study showed that smoking affects several biophysical characteristics of skin. Mainly the thickness of epidermis and dermis was higher and the echodensity of them were lower and the skin was less elastic in smokers. Also nasolabial folds were more obvious in smokers.

Sorencen et al. showed that higher TEWL in smokers’ wound and adjacent skin in comparison with non-smokers 7 days after wounding (19). Increased TEWL by cigarette smoking has also been shown in animal studies (20,21). We also showed that TEWL was higher in smokers skin, although the difference was not statistically significant (21). This can be attributed to the toxic effect of cigarette smoking on SC barrier function (20).

Skin hydration has been shown to be significantly lower in smoker women (22), and the present study showed this in smoker men as well, although the difference wasn’t statistically significant. The responsible mechanism can be the reduced skin blood flow in smokers (23), and the increased TEWL that causes lower skin hydration (24).

Cho et al. showed that erythema index was significantly reduced after smoking cessation (10); this finding is justifiable by the fact that hemoglobin levels are significantly higher in smokers (11). On the other hand it is shown that smoking decreases tissue blood flow (23) and according to this smoking should lighten the skin. Current study did not show a statistically significant difference between smokers’ and non-smokers’ skin erythema. Totally changes of hemoglobin and skin blood flow in smokers have been shown, but more researches are needed to investigate the smoking effect on erythema of skin.

Although melanin content differences were not statistically significant, smokers’ melanin content were higher than non-smokers in all measured sites. This could be due to nicotine effect on melanocytes activity (12, 13).

It is shown that wrinkle formation decreases as skin pH becomes more acidic (25, 26). This study did not show any significant difference in skin pH between smokers and non-smokers, so the increased wrinkle formation caused by smoking could not be attributable to changes in pH.

Sonographic findings showed that thickness of epidermis and dermis was higher and density of epidermis and dermis was lower in smokers in all measured sites, although some of these differences were not statistically significant. The thickness of dermis was significantly higher in smokers’ cheek. Knuutinen et al. also showed that only the thickness of skin on cheeks was significantly higher in smokers. This finding could be explained by the combined effects of smoking and cumulative sun exposure (15).

It has been shown that those who smoke have fewer collagen and elastin fibers in the dermis (27); this finding can justify lower dermis density in smokers which is shown in our study. Fewer collagen and elastin fibers in the dermis cause skin to become slack, hardened and less elastic (27); in current study gross elasticity was significantly lower in smokers on forehead and firmness was higher in smokers in all measured sites, but the differences were not statistically significant.

CSI software showed that surface of nasolabial fold in smokers was higher than non-smokers with statistically significant difference; this finding was in line with previous observations. In a study conducted by Okada et al. on identical twins in which one twin smoked and the other was a non-smoker, the smoking twin had worse scores for nasolabial folds (16). Some other studies have discussed unfavorable smoking effects on nasolabial folds too (28), and overall it is shown that smoking is an important determinant of macroscopic skin wrinkling (29). Smoking can damage the repair mechanisms of skin and affects the extracellular matrix turnover by down regulating collagen and elastin synthesis (16).

Overall, it is recommended that dermatologists could be active participants to encourage patients to quit smoking considering smoking effects on skin (30).

## CONCLUSION

Cigarette smoking could affect the biophysical parameters of skin especially thickness and density of dermis and epidermis and also nasolabial folds surface. Larger sample size is needed to evaluate the effects of smoking on the skin biophysical properties completely.
